# Tracking changes in life-history traits related to unnecessary virulence in a plant-parasitic nematode

**DOI:** 10.1002/ece3.1643

**Published:** 2015-08-13

**Authors:** Philippe Castagnone-Sereno, Karine Mulet, Cathy Iachia

**Affiliations:** 1 UMR1355 Institut Sophia Agrobiotech, INRA06900, Sophia Antipolis, France; 2 UMR7254 Institut Sophia Agrobiotech, University of Nice Sophia Antipolis06900, Sophia Antipolis, France; 3 UMR7254 Institut Sophia Agrobiotech, CNRS06900, Sophia Antipolis, France

**Keywords:** Asexual reproduction, competition, cost of virulence, *Meloidogyne incognita*, plant–nematode interaction

## Abstract

Evaluating trade-offs in life-history traits of plant pathogens is essential to understand the evolution and epidemiology of diseases. In particular, virulence costs when the corresponding host resistance gene is lacking play a major role in the adaptive biology of pathogens and contribute to the maintenance of their genetic diversity. Here, we investigated whether life-history traits directly linked to the establishment of plant–nematode interactions, that is, ability to locate and move toward the roots of the host plant, and to invade roots and develop into mature females, are affected in *Meloidogyne incognita* lines virulent against the tomato *Mi-1.2* resistance gene. Virulent and avirulent near-isogenic lines only differing in their capacity to reproduce or not on resistant tomatoes were compared in single inoculation or pairwise competition experiments. Data highlighted (1) a global lack of trade-off in traits associated with unnecessary virulence with respect to the nematode ability to successfully infest plant roots and (2) variability in these traits when the genetic background of the nematode is considered irrespective of its (a)virulence status. These data suggest that the variation detected here is independent from the adaptation of *M. incognita* to host resistance, but rather reflects some genetic polymorphism in this asexual organism.

## Introduction

Trade-offs in life-history traits play a major role in the evolution of host–parasite interactions (Alizon et al. 2009). Basically, costs arise in the parasite when adaptive change in one life-history trait that helps to exploit the host has a negative impact on another trait of the parasite. Similarly, host resistance to one parasite may confer a selective disadvantage when the resistant host is not challenged by such a parasite. These evolutionary constraints are considered key components of the maintenance of genetic diversity in both the parasites and their hosts (Laine and Tellier 2008).

In plant pathology, the gene-for-gene model predicts that the outcome of the infection of a host plant by a pathogen results from the specific interaction between a given resistance gene in the plant and the corresponding avirulence gene in the pathogen (Flor 1956). Direct or indirect recognition between the products of both the resistance and avirulence genes induces defense responses in the host, and plants or pathogens that lack the resistance or avirulence gene, respectively, are susceptible or virulent (Dangl and Jones 2001). To avoid confusion in terminology, the definition of virulence as used for the purpose of this study will be in direct line with the gene-for-gene concept, that is, ability of the pathogen to overcome the host resistance and establish infection. Evidence for costs of virulence resulting from trade-offs between life-history traits has been documented in many pathosystems involving bacteria (Leach et al. 2001), fungi, and viruses (Laine and Barrès 2013). In gene-for-gene interactions, a mutation from avirulence to virulence in the pathogen is associated with increased fitness on the resistant host, but may in turn becomes disadvantageous on host genotypes lacking the resistance gene. For example, such a cost associated with unnecessary virulence has been reported for the interaction between the rust fungus *Melampsora lini* and flax (Thrall and Burdon 2003), or between the oomycete *Phytophthora infestans* and potato (Montarry et al. 2010). However, contrasting results have also been reported, with the lack of detection of any measurable fitness costs to virulence in, for example, the bean/*Colletotrichum lindemuthianum* or the grape/*Plasmopara viticola* pathosystems (Sicard et al. 2007; Toffolatti et al. 2012).

**Figure 1:**
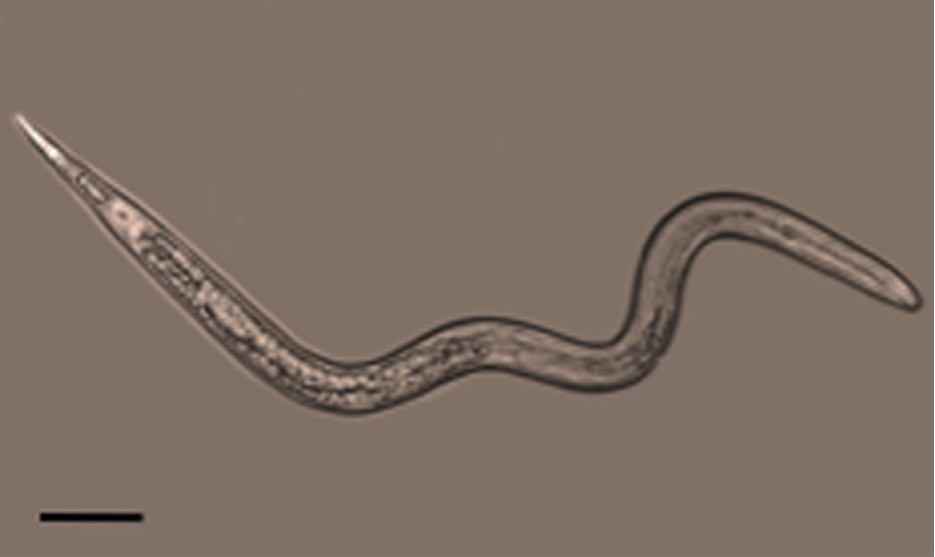
*Meloidogyne incognita* second-stage juvenile. Scale bar = 30 *μ*m.

Root-knot nematodes (RKNs), *Meloidogyne* spp., are considered as one of the most damaging pathogen in the world (Trudgill and Blok 2001). Plant resistance is currently the most efficient strategy for controlling RKNs, although resistance genes may be overcome by virulent populations. For example, virulent populations of *Meloidogyne incognita* (Fig.1), the major RKN species worldwide, can break the *Mi-1.2* and *Me3* resistance genes from tomato and pepper, respectively, that is, can infest and reproduce on resistant cultivars, which raises concern about the durability of resistance (Castagnone-Sereno 2002). In previous experimental studies, we demonstrated that a measurable reproductive fitness cost is associated with unnecessary virulence to both resistance genes in *M. incognita*: when inoculated on susceptible hosts, the reproductive potential of virulent nematodes (i.e., their ability to produce egg masses containing viable J2s able to hatch) is significantly decreased compared to avirulent nematodes (Castagnone-Sereno et al. 2007; Djian-Caporalino et al. 2011). The goal of this study was to investigate whether other life-history traits directly linked to the establishment of the parasitic interaction, that is, ability to locate and move toward the roots of the host plant, and to invade roots and develop into mature females, are also affected in virulent nematodes. The approach used here took plenty advantage of the mitotic mode of reproduction of *M. incognita* that allows the selection of virulent and avirulent near-isogenic lines (NILs) just differing in their capacity to reproduce or not on resistant crops. Indeed, by testing the parasitic behavior of avirulent versus virulent nematode genotypes sharing the same genetic background in single or mixed infections, our study seeks to evaluate the cost of virulence in terms of life-history traits related to infection.

## Materials and Methods

### Plant material

Cultivated tomato, *Solanum esculentum* L., was used as host plant for analyzing the mobility toward roots and the competition behavior of the nematodes. Two cultivars were used, that is, the susceptible cultivar Saint Pierre and the near-isogenic resistant cultivar Piersol carrying the *Mi-1.2* gene (Laterrot 1975).

### Isolation of nucleic acids

Genomic DNA was prepared from ∼200 *μ*L of nematodes according to a standard phenol/chloroform protocol (Sambrook et al. 1989) and stored at −80°C until use. Alternatively, crude extracts from individual nematodes were prepared as described previously (Tigano et al. 2010). Before PCR analyses, genomic DNA samples or crude extracts from individual nematodes were diluted to 5 ng/*μ*L or 20 times, respectively. Quality of DNA was checked by PCR using the *Meloidogyne-*specific primers MelF and MelR (Tigano et al. 2005). In addition, sample identity to the species level was further confirmed using a species-specific SCAR marker as described previously (Randig et al. 2002).

### Evolutionary history of the laboratory system

The experimental system was founded in the laboratory in 1995 based on two *M. incognita* populations sampled from very different geographic regions (Kursk, Russia and Morelos, Mexico, respectively), both avirulent against the tomato *Mi-1.2* resistance gene, in order to minimize the influence of the parasite genetic background on its life-history traits. In addition, to eliminate any potential within-population heterogeneity, a line was raised from each field population, starting from the progeny of a single female carefully dissected from the root tissues along with its egg mass, which was then used to reinoculate a tomato plant. As *M. incognita* reproduces by mitotic parthenogenesis (Triantaphyllou 1985), the second-stage juveniles (J2s) that hatched from each egg mass were considered as a clonal line. From this starting material, sets of replicated lines reared on susceptible or *Mi-1.2*-resistant tomatoes have been maintained since 1996 under controlled conditions, according to described procedures (Jarquin-Barberena et al. 1991; Castagnone-Sereno et al. 2007). Therefore, avirulent and virulent NILs from within a population were considered to vary only in their (a)virulence genes and to be the same at other loci. Considering an average duration of the life cycle of the nematode of about 45 days at 20°C (Ploeg and Maris 1999), the two pairs of avirulent and virulent NILs used in this study had been maintained on Saint Pierre and Piersol, respectively, for ∼120 successive generations. Prior to multiplication, the identity of each line was checked using a species-specific SCAR marker as described previously (Randig et al. 2002).

### Mobility and attraction rate assay

To analyze the ability of the nematodes to locate and move toward the roots of their host plant, we performed an attraction and migration assay in Pluronic gel, modified from a previously described method (Wang et al. 2009). Pluronic F-127 gel is a stable, nontoxic, copolymer that forms a gel at room temperature and a liquid at 15°C and below when the concentration is 20–30%. To make the gel, 23 g of Pluronic F-127 powder (Sigma Life Science, Saint-Quentin Fallavier, France) was added to 90 mL of distilled water and allowed to dissolve with stirring overnight at 4°C. The liquid gel was kept refrigerated at 4°C until use. For each *M. incognita* NIL, 1500 J2s were added into 25 mL 23% Pluronic gel F-127 and gently mixed on ice. The mixture was pipetted into a 12-well culture plate (Corning Inc., New York City, USA) with 700 *μ*L volume (containing ca. 40–45 J2s) in each well. Subsequently, sterile 3-day-old, 1-cm tomato root tips (cultivar Saint Pierre) were placed in the center of the gel, and the plates were incubated 20 min at room temperature for the gel to solidify. Beginning after the solidification of the gel, the distributions of J2s in a 5-mm-diameter area around the root tip were monitored and photographed at 1.5, 3, 4.5, and 6 h. Photographs were taken with a Leica MZ-FL111 stereomicroscope equipped with an AxioCam ICc1 compact digital camera coupled with an AxioVision SE64 Release 4.8 software (Carl Zeiss SAS, Marly le Roi, France). Two replicate plates were analyzed (over time) for each pair of NILs, and the whole set of experiments was repeated four times.

### Development of molecular markers to differentiate avirulent versus virulent nematodes

In a previous study, we identified a set of genes that were differential between avirulent and virulent *M. incognita* NILs (Neveu et al. 2003). Here, four of these genes were selected and used as the priming sites for designing oligonucleotide primers with the Primer3 software (Rozen and Skaletsky 2000). The key parameters set for primer design were as follows: primer length 20–25 bp; PCR amplicon size >450 bp; optimum annealing temperature 55–65°C; GC content 35–65 % with 50% as the optimum. One or two primer pairs per each putative marker were designed, and those that were experimentally validated (i.e., that produce differential amplification patterns between avirulent and virulent nematodes) are given in Table S1. All primers were synthesized by Eurogentec (Seraing, Belgium).

PCR was performed in distilled water in a final volume of 25 *μ*L containing 2 *μ*L genomic DNA (either purified DNA or crude extract from one individual nematode), 1.2 *μ*L dNTP mixtures (10 mmol/L each), 2.5 *μ*L 10× PCR buffer supplemented with MgCl_2_ to a final concentration of 1.5 mmol/L according to the manufacturer's guidelines (MP Biomedicals Taq Core kit, Illkirch-Graffenstaden, France), 1.2 *μ*L primers (10 *μ*mol/L each), and 0.15 *μ*L Taq polymerase (5 U/*μ*L; MP Biomedicals). For the developed molecular markers, thermal cycling conditions were as follows: initial denaturation at 94°C for 3 min, *n* cycles of 94°C for 45 sec, *T*_a_ (annealing temperature) °C for 45 sec, 72°C for 1 min, and a final extension of 72°C for 5 min. Optimal values of *n* and *T*_a_ were adjusted for each primer pair (Table S1). Primer pairs MelF/R and inc-K14-F/R were used as controls to check DNA integrity and species specificity, respectively (Table S1), according to previously published PCR conditions (Randig et al. 2002; Tigano et al. 2005). Amplicons were separated on 2% agarose gels in 1× TBE buffer containing ethidium bromide and visualized under UV light.

### Competition experiments

All experiments were conducted in a growth chamber maintained at 24°C with a 12-h light cycle and a relative humidity of 60–70%. For both pairs of *M. incognita* NILs, susceptible tomatoes (cv. Saint Pierre) were inoculated with avirulent and virulent J2s according to three modalities: one simultaneous co-inoculation (avirulent and virulent nematodes together) and two sequential co-inoculations (avirulent followed by virulent nematodes, and virulent followed by avirulent nematodes, respectively).

Tomato seedlings were grown individually in 50-mL plastic tubes containing steam-sterilized sandy soil. For each modality, 4- to 6-week-old plantlets were inoculated with a water suspension of infective J2s as follows: 100 J2s (50 avirulent + 50 virulent) in the case of simultaneous co-inoculation, or 50 J2s (avirulent or virulent, respectively) followed 24 h later by 50 J2s (virulent or avirulent, respectively) in the case of sequential co-inoculations. Four weeks after inoculation, plants were harvested, their root systems were carefully washed individually with tap water, and mature females were dissected and hand-picked from the root tissues. To check the (a)virulence status of the recovered females, they were individually genotyped using the molecular markers developed previously, according to the PCR conditions described above. Twelve to 24 replicate plants were inoculated for each modality and each pair of nematode NILs, and the experiments were repeated at least twice.

### Statistical analyses

To correct for non-normal distribution, data expressed as counts (in the mobility and attraction rate assay) or percentages (in the competition experiments) were subjected to square root transformation or angular transformation, respectively. For the mobility assay, rates of attraction toward the tomato root were compared between avirulent and virulent nematodes separately at each time point tested and for each pair of NILs. For the competition assay, the relative distributions of avirulent and virulent females in tomato roots were compared separately for each inoculation condition and for each pair of NILs. Pairwise comparisons between means were performed on transformed data with independent-samples Student's *t*-test. Because no significant differences existed between the repeated experiments, results were pooled together for the statistical analyses. All the corresponding computations, and the resulting box plots, were performed using the StatPlus:mac Professional v5 software (AnalystSoft Inc., Vancouver, Canada).

## Results

### Mobility and attraction of nematodes to the roots of their host

**Figure 2:**
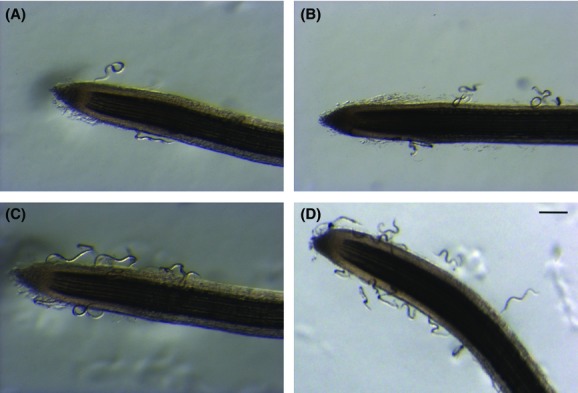
Movement of *Meloidogyne incognita* second-stage juveniles toward tomato root tip in Pluronic gel. Photographs were taken 1.5 (A), 3 (B), 4.5 (C), and 6 h (D) after the solidification of the gel. Nematode genotype used in the experiment = *M. incognita* avirulent NIL from Kursk. Scale bar = 250 *μ*m.

Once the Pluronic gel solidified, nematodes in close vicinity of the tomato root tips were counted at specific times after initiation of the assay. Irrespective of the origin or (a)virulence status of the NILs, the number of *M. incognita* J2s around roots increased with time, from 1.5 to 6 h after the start of the assay (Fig.2). Under our experimental conditions, after longer times, J2s aggregated and formed dense clumps around the root surface, which precluded any further reliable counting (data not shown).

**Figure 3:**
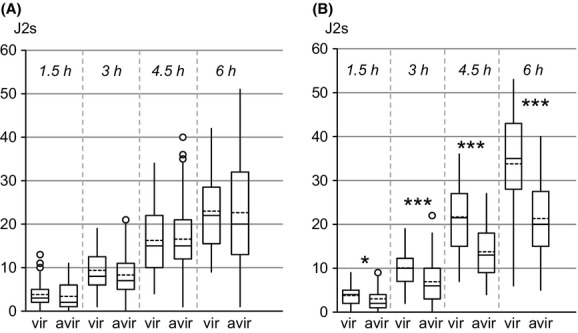
Distribution of *Meloidogyne incognita* second-stage juveniles around tomato root tips in Pluronic gel. (A, B) = avirulent and virulent NILs from Kursk and Morelos, respectively. The ends of each box indicate the first and third quartiles, with the mean and median represented as the dotted and solid line in the box, respectively. The whiskers extending from either end of the box indicate the 10% and 90% quantiles, with outliers given as points. * and *** indicate significant differences in Student's *t*-test (*P *=* *0.038 and *P *<* *0.0001, respectively).

Overall, the number of root tips analyzed at each time point ranged from 65 to 91 and from 71 to 83 for the Kusk and Morelos NILs, respectively. Different rates of attraction/migration toward the roots of their host plant were observed between avirulent and virulent nematodes depending on the origin of the NILs. With the nematodes originating from Kursk, there were no significant differences (*P *>* *0.05) in numbers of avirulent and virulent J2s around the root tips at each time point analyzed (Fig.3A). Conversely, significant differences occurred with nematodes Originally sampled in Morelos: at 1.5 h after assay initiation, more virulent J2s raised root tips compared to avirulent ones (*P *=* *0.038); this trend was reinforced and became highly significant (*P *<* *0.0001) at 3, 4.5, and 6 h (Fig.3B).

### Development of molecular markers that differentiate avirulent versus virulent nematodes

**Figure 4:**
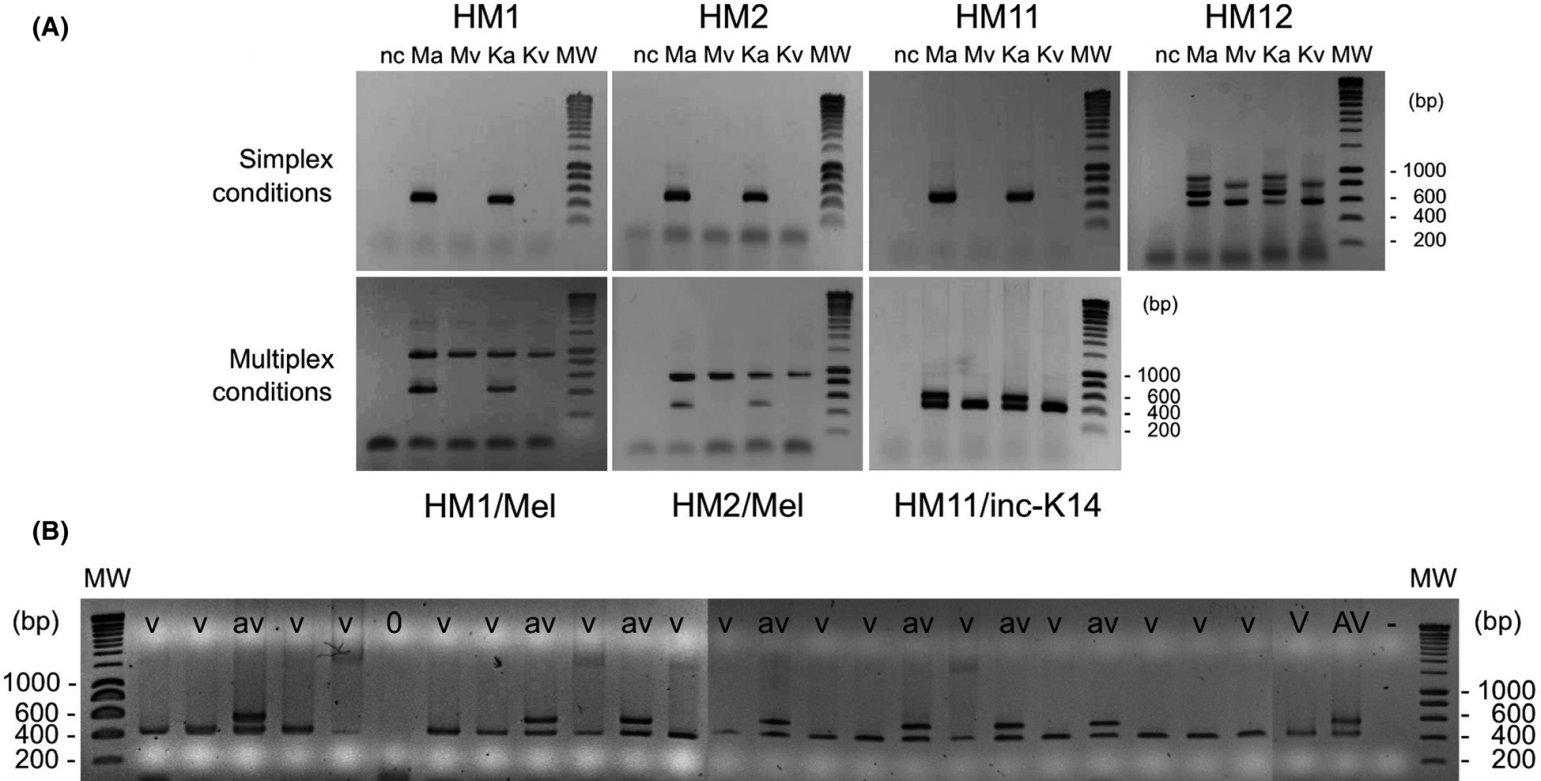
Development of PCR markers that differentiate avirulent versus virulent *Meloidogyne incognita* near-isogenic lines. (A) PCR performed using purified genomic DNA as template, in simplex or multiplex conditions (with *Meloidogyne* spp. or *M. incognita*-specific primer pairs Mel F/R or inc-K14F/R). Ma, Mv and Ka, Kv, avirulent and virulent NILs from Morelos and Kursk, respectively; nc, negative control. (B) An example of genotyping pattern using crude extracts from individual females of the avirulent (av) and virulent (v) NILs from Morelos. V, AV, avirulent and virulent positive controls, respectively; −, negative control. Molecular weights (MWs) are given in base pairs (bp).

In order to be able to genotype avirulent versus virulent nematodes in competition experiments, we developed molecular markers that could be easily used in PCRs. For that purpose, the sequences of four transcript-derived fragments previously identified in a comparative cDNA-AFLP analysis of avirulent and virulent *M. incognita* NILs (Neveu et al. 2003) were used as targets. HM1 and HM11 are pioneer sequences, and HM2 and HM12 encode a ribosomal protein and a SacI homology domain protein, respectively (Neveu et al. 2003). From each of these sequences, using nematode genomic DNAs from both NILs from Kursk and Morelos as templates, primer pairs and PCR conditions were validated, which allowed to unambiguously discriminate virulent nematodes from their avirulent counterparts. Primers specific for HM1, HM2, and HM11 produced very simple amplification patterns, that is, one amplicon with DNA from avirulent nematodes and no band with DNA from virulent nematodes, respectively (Fig.4A). Primers designed from HM12 produced a multiband pattern that was clearly different between avirulent and virulent NILs (Fig.4A). In order to be able to detect false negatives when using the primers specific for HM1, HM2, and HM11 (due to either failure of the PCR or lack of specific DNA template), multiplex conditions with primer pairs MelF/R or inc-K14-F/R were also validated (Fig.4A). Crude DNA extracts from single individual avirulent and virulent females of both pairs of NILs were tested with the primer sets and conditions defined above, and results showed amplification of DNA bands at the expected sizes (Fig.4B), thus demonstrating the reliability and sensitivity of the detection.

### Competition between avirulent and virulent nematodes

**Figure 5:**
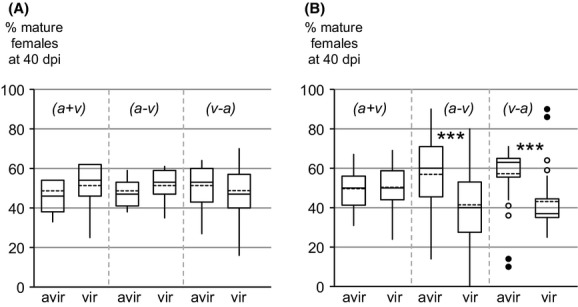
Distribution of *Meloidogyne incognita* mature females in roots of tomato in competition experiments. (A, B) = avirulent and virulent NILs from Kursk and Morelos, respectively. (a + v) = simultaneous co-inoculation; (a − v) and (v − a) = sequential co-inoculations with avirulent followed by virulent nematodes and virulent followed by avirulent nematodes, respectively. The ends of each box indicate the first and third quartiles, with the mean and median represented as the dotted and solid line in the box, respectively. The whiskers extending from either end of the box indicate the 10% and 90% quantiles, with outliers given as points. *** indicates significant differences in Student's *t*-test for the (a − v) and (v − a) sequential co-inoculations with the NILs from Morelos (*P *=* *0.00024 and *P *=* *0.0006, respectively).

Utilizing the molecular markers developed in this study, we were able to assess the relative proportion of avirulent versus virulent nematodes able to infest the tomato roots and further develop into mature females after simultaneous or sequential coinfections. An average number of 27 or 39 females per plant were PCR-analyzed for the Kursk or Morelos NILs, respectively, that represented a total of 5484 individual genotypes determined for the whole experiment. In the case of simultaneous inoculation with equivalent numbers of avirulent and virulent J2s, no significant deviation from the original 50:50 ratio was observed (*P* > 0.05), the same numbers of avirulent and virulent nematodes being recovered from the roots 40 days postinoculation for both pairs of NILs (Fig.5). Conversely, in the case of sequential coinfections, variable proportions of successful nematodes were observed depending on the origin of the NILs. With the NILs from Kursk, the same final pattern as above was detected whatever the order in which avirulent and virulent nematodes were inoculated, that is, no significant deviation from the original 50:50 ratio (*P *>* *0.05) 40 days postinoculation (Fig.5A). Conversely, the final outcome appeared more contrasted with NILs from Morelos, the original 50:50 relation of the avirulent and virulent nematodes being systematically and highly significantly (*P *<* *0.0001) changed in favor of avirulent nematodes independently of the order of inoculations (final ratio of ∼60:40; Fig.5B).

## Discussion

Evaluating life-history trade-offs associated with unnecessary virulence in plant pathogens is generally performed via controlled inoculation experiments on host lacking the resistance factor of interest (Laine and Barrès 2013; Zhan and McDonald 2013). Basically, two types of experimental studies can be designed: (1) single, comparative infections with avirulent or virulent strains (e.g., Thrall and Burdon 2003; Montarry et al. 2010) and (2) competition experiments involving infections with simultaneous mixture of avirulent and virulent strains (e.g., Bahri et al. 2009; Janzac et al. 2010). In addition, in both approaches, working with clonal lineages offers the possibility to compare strains sharing the same genetic background but differing only in their virulence. In the case of plant–nematode interactions, few studies have evaluated the costs of unnecessary virulence on life-history traits. Some data are nevertheless available for avirulent and virulent *M. incognita* isolates that have been compared for their ability to penetrate into roots of susceptible cowpea or tomato genotypes lacking the *Rk* and *Mi-1.2* resistance genes, respectively (Petrillo and Roberts 2005; Melillo et al. 2006). However, to our knowledge, migration of (a)virulent lines toward the host root system and competition between avirulent and virulent lines have not been investigated previously in plant-parasitic nematodes to detect possible virulence trade-offs, and the combination of both experiments, together with the use of NILs resulting from experimental evolution, indeed accounts for the novelty of the present study.

Conversely to predictions from theoretical studies and results of several experimental works, we could not detect here any conserved trade-off between life-history traits and virulence in *M. incognita*. In fact, we surprisingly observed a diversity of response patterns depending on the nematode genotype (i.e., genetic background) considered, the performance of the virulent NILs being either equal, inferior or even superior to that of their respective avirulent counterpart. In particular, the latter in vitro observation that the virulent NIL from Morelos migrated more rapidly toward tomato roots than the avirulent NIL was rather unexpected and opposite to the classical trade-off hypothesis, although such an uncommon positive correlation between virulence and subsequent life-history traits has already been reported, for example, in the oomycete *P. infestans* (Montarry et al. 2010). In previous studies that involved pairs of (a)virulent *M. incognita* NILs from the same geographic origins as those used here, we could demonstrate that a reproductive fitness cost (i.e., a measurable decrease in their ability to produce a progeny) is associated with unnecessary virulence against the tomato *Mi-1.2* or the pepper *Me3* resistance gene, respectively, whatever the nematode origin (Castagnone-Sereno et al. 2007; Djian-Caporalino et al. 2011). The lack of any trade-off between life-history traits related to invasion of the host roots and virulence, as opposite to reproductive potential, thus indicates that the various components that determine the overall nematode parasitic success are independently affected by selection. Several mechanisms may account for the lack of a cost for unnecessary virulence in *M. incognita*. The most parsimonious hypothesis is that such a cost is indeed absent (as in the oomycete *Pl. viticola*; Toffolatti et al. 2012) or so low that the experimental design used here was not sensitive/appropriate enough to detect it (Zhan and McDonald 2013). Assuming that fitness results from the combination of multiple basic components, which is very likely in the case in multicellular parasites like nematodes, it is not unrealistic to consider that the specific life-history traits investigated in the present study may not be negatively impacted by the virulence status of the NILs. Alternatively, compensatory mechanisms may act to reduce the penalties associated with unnecessary virulence (Luna et al. 2009; Montarry et al. 2010), and/or the relative role of a particular avirulence factor in fitness may vary in different genetic backgrounds (Shan et al. 2000). Separately or in combination, such mechanisms may be relevant to explain the diversity of responses observed here in relation to the genetic background of the NILs. In part due to their complexity, the mobility experiments were conducted here on one single tomato genotype, although different host genetic backgrounds may have induced variability in the nematode behavior. However, such variability was not observed when testing different tomato cultivars for infection with *M. hapla*, *M. incognita,* and *M. javanica* in the same experimental conditions (Wang et al. 2009). This result indicates that the tomato genotype does not act upon the nematode behavior, which somehow supports the validity of our own results.

Coinfections of individual hosts by multiple eukaryotic parasite species are very commonly observed in natural populations, and an abundant literature has been focusing on the epidemiological and evolutionary consequences of competition in the fields of parasitology and plant pathology (reviewed in Alizon et al. 2013; Viney and Graham 2013; Zhan and McDonald 2013). In the case of plant–nematode interactions, the outcome of interspecific competition experiments involving RKNs together with other nematode species has been extensively documented (Aryal et al. 2011; Diez et al. 2003; Melakeberhan and Dey 2003; Brinkman et al. 2005). However, reports of competition studies at the infraspecific level, that is, involving different isolates of the same RKN species, are scarce, probably because of the lack of reliable and user-friendly molecular markers to differentiate such otherwise undistinguishable nematode isolates. To overcome this drawback, we have developed here a set of PCR primers that provided a clear differentiation between *M. incognita* avirulent and virulent NILs, using polymorphic cDNA fragments identified in a previous study (Neveu et al. 2003) as targets. With these molecular markers, we could unambiguously evaluate the outcome of simultaneous or sequential coinfections of two pairs of avirulent and virulent NILs on susceptible hosts, which constitutes, to our knowledge, the first study of intraspecific competition in RKNs.

When susceptible tomatoes were simultaneously inoculated with an equivalent amount of avirulent and virulent J2s, the final outcome was an equilibrated successful coinfection, with no deviation from the 50:50 ratio in the number of avirulent and virulent nematodes able to produce a progeny on the root system. The same observation was obtained with NILs originating from Kursk and Morelos, although the ability of the virulent line from Morelos to migrate toward the plant roots was higher compared to its avirulent counterpart. This result, however, is not always the rule in the case of plant–pathogen interactions. For example, in the case of the wheat pathogen *Puccinia striiformis* f.sp. *tritici*, pairwise competition experiments showed variations in the relative frequencies of avirulent versus virulent isolates able to infect host plants lacking the corresponding resistance gene, with isolates bearing the additional virulence generally less competitive, although the opposite reaction (i.e., strong competitive advantage) was observed in the case of the virulence factor *vir9* (Bahri et al. 2009). These contrasting results indicate that predicting the outcome of a competition experiment involving avirulent and virulent plant pathogens is simply not possible a priori in the current state of knowledge. In the case of sequential co-inoculations, a different outcome was observed when NILs originating from Kursk and Morelos were considered, respectively. Various host–parasite systems have suggested that prior inoculation provides a competitive advantage, because the second inoculated strain has to face a depletion of host resources and the activation of host defenses (Weeds et al. 2000; Read and Taylor 2000). Here, such a competitive advantage linked to prior residency was not observed, except when the avirulent NIL from Morelos was inoculated 24 h before its virulent counterpart. Again, the genetic background of the *M. incognita* lines appears to be a major factor that impacts the final issue in intraspecific competition. Conversely to other biological models (Staves and Knell 2010; Bashey et al. 2012), no relationship could be found here between virulence and competitive ability.

Overall, our study revealed a lack of life-history trade-offs associated with unnecessary virulence in *M. incognita* with respect to its ability to locate and move toward the root of the host plant, and to invade it in order to develop into mature females. Conversely, some variability in these traits was found when the genetic background of the nematodes was considered independently from their (a)virulence status. Both isolates from Kursk and Morelos were sampled on tomato, introduced in the nematode living collection of the laboratory at the same time, and exclusively reared on tomato since then. In addition, previous molecular data showed a very low level of neutral genetic diversity between them (Semblat et al. 2001; Neveu et al. 2003). However, previous elements of their relative evolutionary history remain largely unknown. In particular, as *M. incognita* is preferentially found under (sub)tropical climates, the Kursk isolate might be the result of a (recent) introduction in Russia and thus might have suffered some genetic bottleneck that affected its genetic diversity. Clearly, further studies are needed to accurately evaluate the extent of polymorphism in life-history traits in relation to the genotype of the isolates.

The *Mi-1.2* gene is the only RKN resistance gene introgressed in all the commercially available resistant tomato cultivars worldwide and has been successfully used to control RKNs for more than 60 years, even if cases of virulent populations have been reported in major tomato-producing areas (Williamson and Roberts 2009). From this point of view, *Mi-1.2* should be considered as a very durable gene, and such durability may appear paradoxical when considering that no cost is associated with virulence in the nematode. In fact, at least two arguments may be advanced to explain the relative poor frequency of *Mi-1.2* virulent *M. incognita* populations in agro-ecosystems. First, the pathogenic pressure exerted by RKNs on resistant crops is rather poor, because of the biological characteristics of these telluric organisms (i.e., their limited capacity of active movement and propagule production compared to fungal spores or bacteria). Second, the lack of cost in life-history traits as evidenced in this study may be in part compensated by the reproductive cost associated with *M. incognita* virulence against the tomato *Mi-1.2* resistance gene (Castagnone-Sereno et al. 2007; Djian-Caporalino et al. 2011), which should result in a dilution of virulent genotypes in case of sympatric avirulent and virulent populations associated with susceptible plants. Clearly, expanding the understanding of the evolutionary forces driving the diversity of the plant–nematode interactions appears of outstanding importance to address crucial plant protection issues.

## Supporting Information

**Table S1.** List of primers used in this study.
