# Supplementary material for: Tracking changes in life-history traits related to unnecessary virulence in a plant-parasitic nematode
Source: Ecol Evol. 2015 Aug 13;5(17):3677–86. doi: 10.1002/ece3.1643 (PMC4567871; doi:10.1002/ece3.1643)
Supplement: Supplementary file 1 — Table S1. List of primers used in this study. [file ece30005-3677-sd1.docx]

**Supplementary information**

## Table S1 - List of primers used in this study

**Primer 5' to 3' sequence Annealing Number Amplicon(s) Reference**

**name temperature of cycles size**

HM1.F2 ATAGAATGTCCAACAAGTAGAGGC 64°C 30 ~480 bp this study

HM1.R2 TGGCAGAAAGTATCGTGTGAT

HM2.F ATGTTGGACGCACTGAATTTT 61°C 36 ~500 bp this study

HM2.R AGCACGTCCCAACGCTGCCTT

HM11.F2 TTAGCGCCAACGATGATTAGA 66°C 34 ~550 bp this study

HM11.R2 CATGGCCATTCCCTCCTATT

HM12.F2 GGAACTGTTTAGGGGTATTGGTAT 58°C 41 ~580 to 900 bp^a^ this study

HM12.R2 AGGGGGAGGGGGAGTCAATAT

inc-K14-F GGGATGTGTAAATGCTCCTG 64/62°C^b^ 32 399 bp Randig et al., 2002

inc-K14-R CCCGCTACACCCTCAACTTC

MelF TACGGACTGAGATAATGGT 50°C 34 901 bp Tigano et al., 2005

MelR GGTTCAAGCCACTGCGA

^a^Four bands (~580/700/800/900 bp) or two bands (~580/800 bp) in avirulent or virulent nematodes, respectively.

^b^In simplex or multiplex condition, respectively.
